# Racial and Socioeconomic Differences in Heart Failure Hospitalizations and Telemedicine Follow-up During the COVID-19 Pandemic: Retrospective Cohort Study

**DOI:** 10.2196/39566

**Published:** 2022-11-28

**Authors:** Zachary Hughes, Julia Simkowski, Parry Mendapara, Nicolas Fink, Sparsh Gupta, Quentin Youmans, Sadiya Khan, Jane Wilcox, R Kannan Mutharasan

**Affiliations:** 1 Department of Medicine Northwestern University Feinberg School of Medicine Chicago, IL United States; 2 Northwestern University Feinberg School of Medicine Chicago, IL United States; 3 Division of Cardiology Northwestern University Feinberg School of Medicine Chicago, IL United States; 4 Division of Preventive Medicine Northwestern University Feinberg School of Medicine Chicago, IL United States

**Keywords:** heart failure, disparities, disparity, SARS-CoV-2, Coronavirus, pandemic, COVID-19, hospitalization, telemedicine, heart disease, heart, socioeconomic, cardiology, cardiac, hospital admission, intensive care unit, ICU admission, mortality, inequality, inequalities, minority, socioeconomic, health disparity, racial, ethnic

## Abstract

**Background:**

Low rates of heart failure (HF) hospitalizations were observed during the 2020 peak of the COVID-19 pandemic. Additionally, posthospitalization follow-up transitioned to a predominantly telemedicine model. It is unknown whether the shift to telemedicine impacted disparities in posthospitalization follow-up or HF readmissions.

**Objective:**

The aim of this paper is to determine whether the shift to telemedicine impacted racial and ethnic as well as socioeconomic disparities in acute decompensated heart failure (ADHF) follow-up and HF readmissions. We additionally sought to investigate the impact of the COVID-19 pandemic on the severity of ADHF hospitalizations.

**Methods:**

This was a retrospective cohort study of HF admissions across 8 participating hospitals during the initial peak of the COVID-19 pandemic (March 15 to June 1, 2020), compared to the same time frame in 2019. Patients were stratified by race, ethnicity, and median neighborhood income. Hospital and intensive care unit (ICU) admission rates, inpatient mortality, 7-day follow-up, and 30-day readmissions were assessed.

**Results:**

From March 15, 2019, to June 1, 2020, there were 1162 hospitalizations for ADHF included in the study. There were significantly fewer admissions for ADHF in 2020, compared with 2019 (442 vs 720; *P*<.001). Patients in 2020 had higher rates of ICU admission, compared with 2019 (15.8% vs 11.1%; *P*=.02). This trend was seen across all subgroups and was significant for patients from the highest income quartile (17.89% vs 10.99%; *P*=.02). While there was a trend toward higher inpatient mortality in 2020 versus 2019 (4.3% vs 2.8%; *P*=.17), no difference was seen among different racial and socioeconomic groups. Telemedicine comprised 81.6% of 7-day follow-up in 2020, with improvement in 7-day follow-up rates (40.5% vs 29.6%; *P*<.001). Inequities in 7-day follow-up for patients from non-Hispanic Black racial backgrounds compared to those from non-Hispanic White backgrounds decreased during the pandemic. Additionally, those with telemedicine follow-up were less likely to be readmitted in 30 days when compared to no follow-up (13.8% vs 22.4%; *P*=.03).

**Conclusions:**

There were no major differences in HF ICU admissions or inpatient mortality for different racial and socioeconomic groups during the COVID-19 pandemic. Inequalities in 7-day follow-up were reduced with the advent of telemedicine and decreased 30-day readmission rates for those who had telemedicine follow-up.

## Introduction

Acute decompensated heart failure (ADHF) is the leading hospital discharge diagnosis in the United States [1]. However, during the initial peak of the global COVID-19 pandemic, there was an unprecedented decrease in ADHF hospitalizations. In Europe, reports from individual hospitals as well as national data registries have shown reductions in heart failure (HF) admissions ranging from 30% to 50% [2,3]. Several institutions in the United States have shown similar decreases in HF admissions [4,5]. Despite lower admission rates, when patients do present, they are more ill with higher New York Heart Association class and more severe peripheral edema [6]. The global COVID-19 pandemic has not only affected the rates and severity of ADHF hospitalizations, but also postdischarge follow-up. Early follow-up, especially within 7 days after discharge, has been associated with better outcomes and reduced 30-day readmissions for patients with ADHF [7,8]. With wide-ranging stay-at-home orders to prevent the spread of infection, many US health care systems reduced in-person clinic visits in favor of telemedicine phone and video interactions. Several professional societies, including the Heart Failure Society of America, have released statements in support of telemedicine visits, though it has yet to be seen as an effective tool in reducing 30-day readmission rates [9].

While the COVID-19 pandemic has had many different effects on the health care system, one constant thread has been the disproportionate toll the virus has had on patients who self-identify with racial and ethnic minority groups, as well as patients who live in lower-income neighborhoods [10]. In Chicago, Black individuals were burdened with more than 50% of the COVID-19 cases and 70% of COVID-19 deaths despite representing only 30% of the city’s population [11]. Parallel disparities in HF outcomes associated with race, ethnicity, and socioeconomic status have been seen. Patients with HF who self-identify as Black or are residents of lower-income neighborhoods have worse health status and higher rates of mortality when compared to the general population [12]. These at-risk groups already struggle with access to care, and the COVID-19 pandemic may further exacerbate these disparities. Currently, there are no studies investigating the impact of the transition to telemedicine on the incidence of HF readmissions and early posthospitalization follow-up for patients of different racial, ethnic, and socioeconomic backgrounds. It is also unknown whether the COVID-19 pandemic affected the incidence or severity of ADHF admissions for patients of varying minority groups. Therefore, the objective of this study is twofold: (1) determine whether the shift to telemedicine impacted racial and ethnic as well as socioeconomic disparities in ADHF follow-up and HF readmissions, and (2) determine the impact of the COVID-19 pandemic on the severity of ADHF hospitalizations.

## Methods

### Patient Selection

This was a retrospective cohort study derived from patients who were hospitalized for ADHF within the Northwestern Memorial Healthcare (NMHC) system. The NMHC system comprises over 200 sites and 10 hospitals that provide health care to a varying and diverse population throughout the city of Chicago and the surrounding metropolitan area. Overall, 8 hospitals were included that collected data on patients who were hospitalized in 2019 and 2020. Two hospitals that did not collect data in 2019 were excluded.

**Figure 1:**
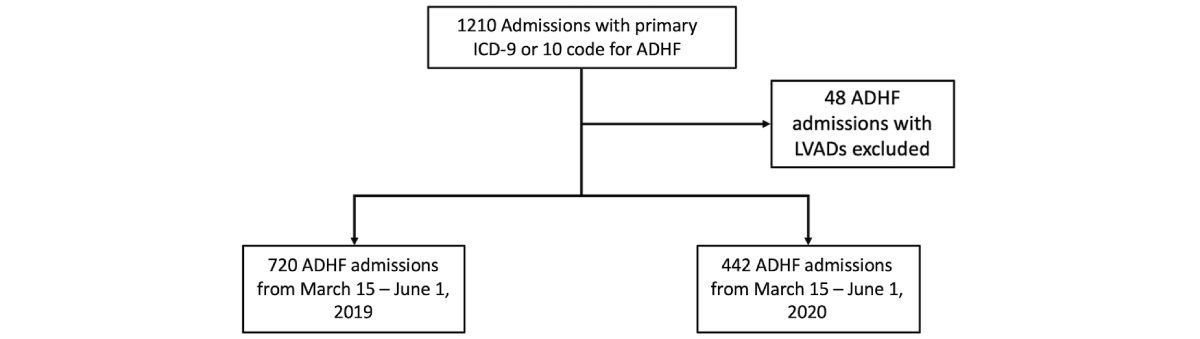
Patient selection. ADHF: acute decompensated heart failure; ICD: International Classification of Diseases; LVAD: left ventricular assist device.

Patient data were obtained via the Northwestern Medicine Enterprise Data Warehouse. The latter provides a single comprehensive repository of all clinical and research data across all NMHC facilities. Included in this retrospective cohort study were adults aged 18 years and older who were hospitalized with the International Classification of Diseases (ICD), ninth revision (ICD-9: 402.01, 402.11, 402.91, 404.01, 404.03, 404.11, 404.13, 404.91, 404.93, and 428.0-428.9) or tenth revision, ICD-10 (I11.0, I13.0, I13.2, and I50.0-I50.9) primary diagnosis of ADHF (Figure 1). Patients with left ventricular assist devices were excluded. For those who were hospitalized with a primary ICD diagnosis of ADHF, patient characteristics including age, race, ethnicity, zip code, BMI, weight, most recent left ventricular ejection fraction determined by echocardiography, as well as the comorbidities hypertension, chronic obstructive pulmonary disease, and type 1 or type 2 diabetes mellitus were obtained via chart review. During the peak of the COVID-19 pandemic, a stay-at-home order was placed for the city of Chicago and the state of Illinois from March 19, 2020, to June 3, 2020. This order required citizens to self-isolate at home except for essential needs, such as grocery shopping or seeking medical care. Therefore, we selected patients hospitalized for ADHF from March 15, 2020, to June 1, 2020, and compared these patients to those who were admitted from March 15, 2019, to June 1, 2019.

### Statistical Analysis

The primary objective of this study was to investigate the effect of the COVID-19 pandemic on both acuity of ADHF hospitalizations as well as changes in posthospitalization follow-up after the implementation of telemedicine. The endpoints assessed included the rate of inpatient mortality, predicted inpatient mortality calculated by validated risk scores, incidence of intensive care unit (ICU) admission, posthospitalization follow-up within 7 days, and 30-day readmission rates. Predicted in-hospital mortality was determined using Get With the Guidelines—Heart Failure (GWTG-HF) as well as the Organized Program to Initiate Lifesaving Treatment in Hospitalized Patients with Heart Failure (OPTIMIZE-HF) risk scores. Both the GWTG-HF score and the OPTIMIZE-HF score are successful predictors of in-hospital mortality for both patients with reduced HF and preserved left ventricular ejection fraction [13,14]. GWTG-HF and OPTIMIZE-HF risk scores were calculated using the variables as previously reported [13,14]. For both risk scores, a patient’s score is obtained by summing points assigned to the value of each predictor. The values of the score are between 0 and 100, with higher scores correlating to a higher-percent predicted inpatient mortality [13,14]. Follow-up was codified as either in-person or telemedicine as documented in the electronic medical record. Telemedicine follow-up included both phone as well as video encounters as documented by the outpatient primary provider in the electronic medical record.

Results were stratified by race and ethnicity as well as median neighborhood income quartile. Patients were divided into income quartiles based on their zip code’s median household income using data from the US Census Bureau American Community Survey 2010-2014. Income quartile 1 represents the lowest median household income while income quartile 4 represents the highest.

Data are presented as mean values with SD if normally distributed and median with interquartile range if skewed. Categorical data are presented as frequencies and percentages. Baseline characteristics and outcomes were compared between the 2019 historical control cohort and the 2020 peak COVID-19 pandemic cohort, using Student *t* tests and χ^2^ tests where appropriate. *P*<.05 (2‐sided test) was considered significant. All statistical tests and analyses were performed with Stata, version 16 (StataCorp).

### Ethics Approval

This study was approved by the institutional review board at Northwestern University (IRB approval number: STU00213213), and the procedures followed were in accordance with the with the Helsinki Declaration of 1975, as revised in 2000. All patient data included within the study have been deidentified.

## Results

Overall, there were 1162 hospitalizations for ADHF between the 2 cohorts. There were significantly fewer admissions for ADHF in 2020 compared with 2019 (442 vs 720; *P*<.001). Baseline patient characteristics are presented in Table 1.

Patients admitted in 2020 were more likely to have chronic obstructive pulmonary disease (26.2% vs 19.8%; *P*=.01) and had a higher percentage admitted from income quartile 3 (20.1% vs 10.6%; *P*<.001) as well as fewer from income quartile 2 (4.5% vs 7.9%; *P*=.02). There were significantly more patients with non-Hispanic White racial backgrounds (59.7% vs 70.6%; *P*<.001*)* admitted in 2020 with a coinciding decrease in non-Hispanic Black admissions (27.8% vs 19.4%; *P*=.001).

**Figure 2:**
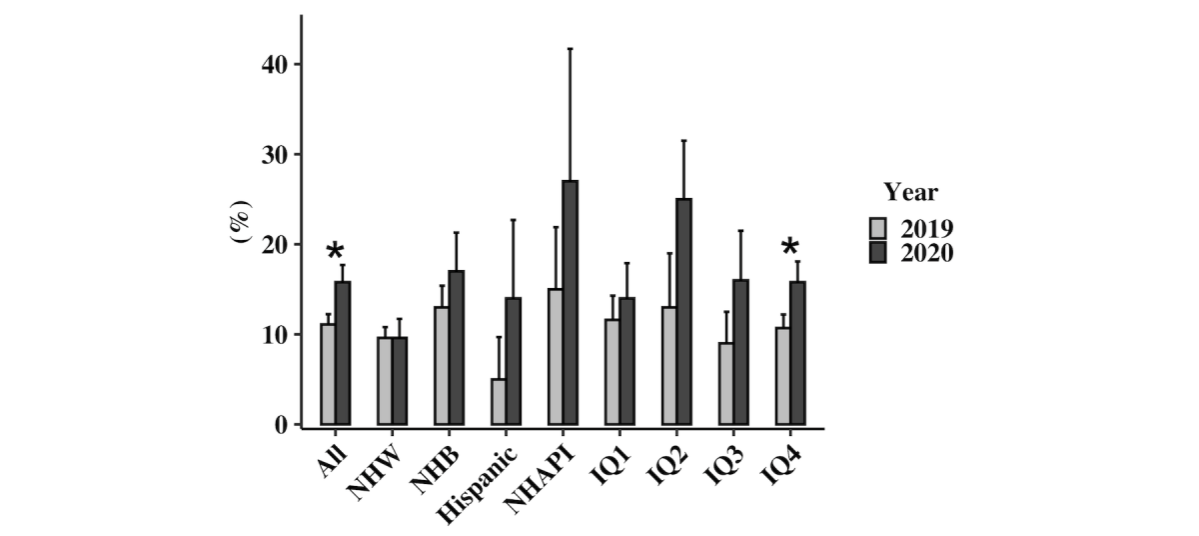
Intensive care unit admission rates 2019 vs 2020. IQ: income quartile; NHAPI: non-Hispanic Asian or Pacific Islander; NHB: non-Hispanic Black; NHW: non-Hispanic White.

Inpatient outcomes for those hospitalized with ADHF are reported in Table 2 as well as Figure 2.

A greater proportion of patients were admitted to the ICU in 2020 compared to 2019 (15.8% vs 11.1%; *P*=.02). Patients from income quartile 4 (15.8% vs 10.7%; *P*=.05) had greater rates of ICU admissions in 2020 compared to 2019. Though not statistically significant, there was a trend toward higher mortality for those admitted in 2020 compared to 2019 (4.3% vs 2.8%; *P*=.17). There was no difference in predicted inpatient mortality between the 2019 and 2020 cohorts or any of the racial or socioeconomic subgroups.

**Figure 3:**
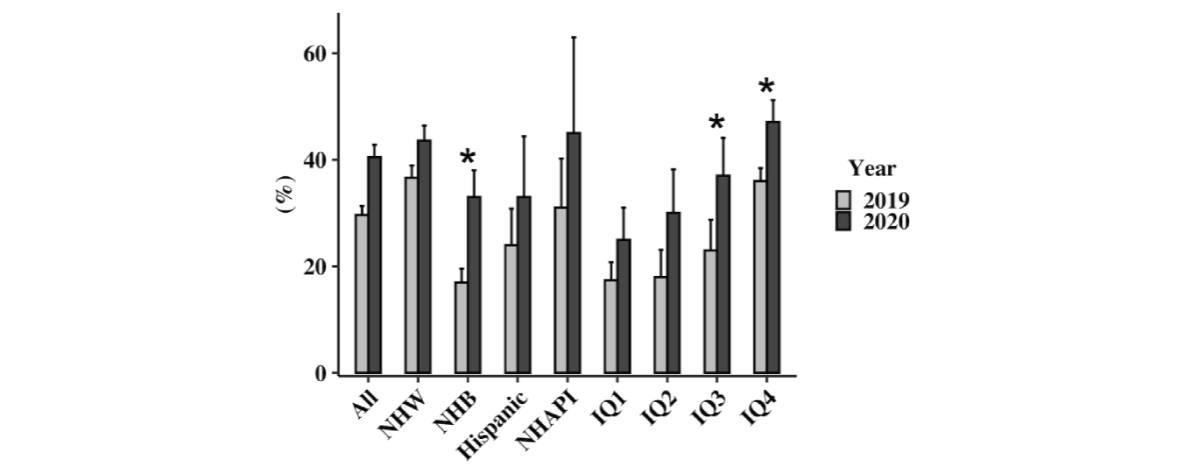
Seven-day follow-up rates, 2019 versus 2020. IQ: income quartile; NHAPI: non-Hispanic Asian or Pacific Islander; NHB: non-Hispanic Black; NHW: non-Hispanic White.

**Figure 4:**
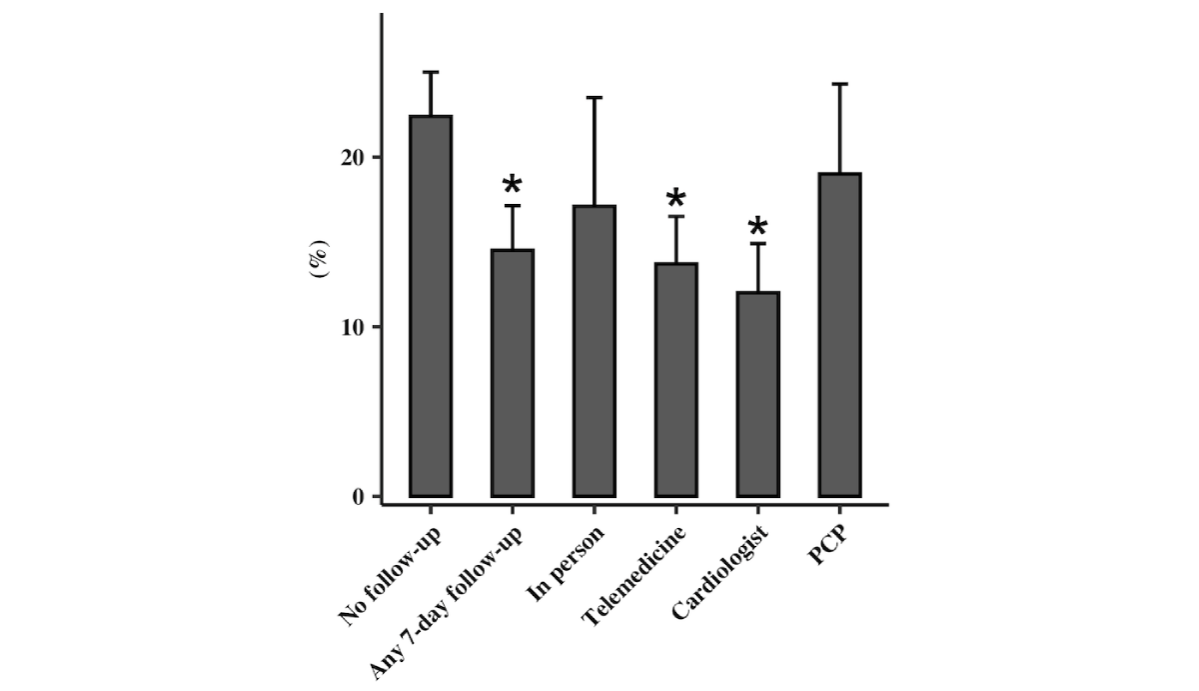
Thirty-day readmission rates in 2020. PCP: primary care physician.

Follow-up and readmission data are reported in Tables 3 and 4, as well as Figures 3 and 4.

In 2020, 81.6% of 7-day follow up was conducted via telemedicine as compared to 0% in 2019 (*P*<.001). Moreover, 142 (97.2%) of the telemedicine follow-up encounters were conducted via phone while only 4 (2.8%) were conducted by video. A greater proportion of patients had successful 7-day follow-up in 2020 compared to 2019 (40.5% vs 29.6%; *P*<.001). All racial, ethnic, and socioeconomic groups saw a trend toward improved 7-day follow-up with patients who had non-Hispanic Black racial backgrounds (33% vs 17%; *P*=.003) meeting statistical significance as well as those from income quartiles 3 (37% vs 23%; *P*=.05) and 4 (47.1% vs 36.0%; *P*=.004). Female patients also saw significant improvements in follow-up with the implementation of telemedicine as compared to 2019 (41.9% vs 24.7%; *P*<.001). In 2020, patients who received follow-up within 7 days of hospital discharge were significantly less likely to be readmitted in 30 days when compared to those who had no follow-up (14.5% vs 22.4%; *P*=.04). Patients who received 7-day follow-up via telemedicine (13.8% vs 22.4%; *P*=.03) were also less likely to be readmitted in 30 days when compared to no follow-up.

**Table 1:** Baseline characteristics.

Characteristics		2019 (n=720)	2020 (n=442)	*P* value
Age (years), mean (SD)		73.1 (0.9)	72.4 (0.7)	.44
Gender (male), n (%)		378 (52.5)	232 (52.4)	.48
**Race or ethnicity, n (%)**				
	NHW^a^	429 (59.7)	312 (70.6)	<.001
	NHB^b^	200 (27.8)	86 (19.4)	.001
	Hispanic	45 (6.2)	27 (6.1)	.94
	NHAPI^c^	26 (3.6)	11 (2.5)	.30
	Other	19 (2.7)	6 (1.4)	.87
BMI (kg/m2), mean (SD)		32.49 (0.8)	32.60 (0.6)	.89
Weight (lbs), mean (SD)		200.5 (3.9)	203.1 (3.2)	.38
COPD^d^, n (%)		142 (19.8)	100 (26.2)	.01
DM2^e^, n (%)		326 (45.4)	205 (45.7)	.89
HTN^f^, n (%)		626 (87.1)	349 (85.7)	.47
**Income quartile, n (%)**				
	IQ1^g^	139 (19.3)	73 (16.5)	.23
	Q2	57 (7.9)	20 (4.5)	.02
	Q3	77 (10.6)	89 (20.1)	<.001
	Q4	447 (62.1)	259 (58.6)	.24
LVEF^h^ (<40%), n (%)		249 (34.6)	159 (35.9)	.68
Average percentage of LVEF, mean (SD)		47.5 (0.68)	47.1 (1.23)	.76

^a^NHW: non-Hispanic White.

^b^NHB: non-Hispanic Black.

^c^NHAPI: non-Hispanic Asian or Pacific Islander.

^d^COPD: chronic obstructive pulmonary disease.

^e^DM2: type 2 diabetes mellitus.

^f^HTN: hypertension.

^g^IQ: income quartile.

^h^LVEF: left ventricular ejection fraction.

**Table 2:** Inpatient outcomes.

Variable			Value				
			2019		2020		*P* value
**ICU^a^ admissions, n (%)**							
	Total		80 (11.1)	70 (15.8)		.02	
	**Sex**						.10
		Male	44 (11.6)	38 (16.3)			
		Female	36 (10.6)	32 (15.2)			
	**Race or ethnicity**						
		NHW^b^	41 (9.6)	30 (9.6)		.98	
		NHB^c^	26 (13)	15 (17)		.33	
		Hispanic	3 (5)	4 (14)		.21	
		NHAPI^d^	4 (15)	3 (27)		.62	
	**Income quartile**						
		IQ1^e^	16 (11.6)	10 (14)		.61	
		IQ2	7 (13)	5 (25)		.21	
		IQ3	7 (9)	14 (16)		.18	
		IQ4	49 (10.7)	41 (15.8)		.05	
**Mortality, n (%)**							
	Total		20 (2.8)	19 (4.3)		.17	
	**Sex**						
		Male	10 (2.8)	10 (4.3)		.22	
		Female	10 (2.8)	9 (4.3)		.23	
	**Race or ethnicity**						
		NHW	14 (1.9)	16 (5.1)		.20	
		NHB	3 (1.5)	1 (1)		.83	
		Hispanic	2 (4)	1 (4)		.93	
		NHAPI	0 (0)	1 (11)		.08	
	**Income quartile**						
		IQ1	3 (2.2)	2 (3)		.79	
		IQ2	2 (4)	1 (5)		.64	
		IQ3	2 (3)	4 (4)		.77	
		IQ4	12 (2.7)	13 (5.0)		.11	
**GWTG-HF^f^ score**							
	Total		40.86	40.74		.82	
	**Sex**						
		Male	41.67	41.78		.89	
		Female	39.95	39.68		.72	
	**Race or ethnicity**						
		NHW	42.99	42.24		.22	
		NHB	35.17	34.59		.60	
		Hispanic	40.81	36.25		.051	
		NHAPI	44.50	42.82		.51	
	**Income quartile**						
		IQ1	36.68	38.27		.26	
		IQ2	40.86	38.15		.33	
		IQ3	40.51	39.59		.47	
		IQ4	42.18	42.00		.79	
**OPTIMIZE-HF^g^ score**							
	Total		34.49	34.05		.39	
	**Sex**						
		Male	35.35	35.05		.68	
		Female	33.53	33.06		.49	
	**Race or ethnicity**						
		NHW	35.49	35.00		.43	
		NHB	32.17	31.35		.43	
		Hispanic	32.71	30.52		.26	
		NHAPI	37.88	36.63		.20	
	**Income quartile**						
		IQ1	32.69	33.57		.57	
		IQ2	34.12	33.10		.70	
		IQ3	34.12	33.26		.49	
		IQ4	35.08	34.54		.39	

^a^ICU: intensive care unit.

^b^NHW: non-Hispanic White.

^c^NHB: non-Hispanic Black.

^d^NHAPI: non-Hispanic Asian or Pacific Islander.

^e^IQ: income quartile.

^f^GWTG-HF: Get With the Guidelines—Heart Failure.

^g^OPTIMIZE-HF: Organized Program to Initiate Lifesaving Treatment in Hospitalized Patients with Heart Failure.

**Table 3:** Posthospitalization follow-up.

Variable			Value					
			2019, n (%)		2020, n (%)		*P* value	
Total			213 (29.6)		179 (40.5)		<.001	
**Sex**								
	Male	129 (33.9)		87 (39.2)		.19		
	Female	84 (24.7)		92 (41.8)		<.001		
**Race or ethnicity**								
	NHW^a^	157 (36.6)		136 (43.6)		.05		
	NHB^b^	34 (17)		28 (33)		.003		
	Hispanic	11 (24)		9 (33)		.41		
	NHAPI^c^	8 (31)		5 (45)		.39		
**Income quartile**								
	IQ1^d^	24 (17.4)		18 (25)		.19		
	IQ2	10 (18)		6 (30)		.26		
	IQ3	18 (23)		33 (37)		.05		
	IQ4	161 (36)		122 (47.1)		.004		
Telemedicine			0 (0)		146 (81.6)		<.001	
Cardiologist			131 (61.5)		125 (69.8)		.09	
Primary care provider			82 (38.5)		54 (30.2)		.09	

^a^NHW: non-Hispanic White.

^b^NHB: non-Hispanic Black.

^c^NHAPI: non-Hispanic Asian or Pacific Islander.

^d^IQ: income quartile.

**Table 4:** Thirty-day readmission rates by follow-up type.

Year and follow-up type		Value	
		N (%)	*P* value
**2019**			
	No follow-up	127 (25)	Reference
	Any 7-day follow-up	46 (21.6)	.29
	Cardiologist	30 (22.9)	.61
	Primary care provider	15 (19)	.22
**2020**			
	No follow-up	59 (22.4)	Reference
	Any 7-day follow-up	26 (14.5)	.04
	Cardiologist	15 (12)	.03
	Primary care provider	10 (19)	.53
	Telemedicine	20 (13.7)	.03
	In person	6 (17.1)	.47

## Discussion

### Principal Findings

This study investigated differences in ADHF hospitalizations, inpatient outcomes, posthospitalization follow-up, and 30-day readmissions for patients of varying racial, ethnic, and socioeconomic backgrounds during the COVID-19 pandemic. The principal findings of our study are that patients with ADHF, admitted during the COVID-19 pandemic, (1) were more likely to be admitted to the ICU, though there were no major differences in ICU admission rates among different racial and ethnic minorities or neighborhood income levels; (2) had lower rates of 30-day readmissions with telemedicine follow-up within 7 days of discharge; and (3), perhaps most importantly, had a reduction in follow-up rate disparities and increased rates of 7-day follow-up with the advent of telemedicine. Though previous studies have reported on ADHF admissions and outcomes during the COVID-19 pandemic, to our knowledge, our study is the first to investigate racial, ethnic, and socioeconomic differences for patients with HF during the acute hospitalization. It is also the first study to assess the impact of telemedicine on disparities in ADHF posthospitalization follow-up and 30-day readmission rates.

### Comparison With Prior Work

Our study supports prior findings of reduced admissions for ADHF seen throughout the United States and Europe with the onset of the COVID-19 pandemic [2-6]. This phenomenon is likely secondary to a complex interplay of multiple public health and social factors. It is possible that fear of acquisition of COVID-19 associated with the medical environment and strict social isolation precautions placed by local and national authorities may have prejudiced patients to defer pursuit of medical care or attempt to self-manage care at home. The decreased overall admissions and concurrent increase in ICU admission rates seen in 2020 compared to 2019 supports the notion that patients may have delayed care until their disease status was more progressed.

The COVID-19 pandemic has had an unequal toll on patients from racial and ethnic minorities and those from lower income neighborhoods. Self-identified Black patients and those from poorer neighborhoods who contracted SARS-CoV-2 were more likely to be hospitalized and have worse inpatient outcomes when compared to White patients and those from more affluent areas [15,16]. Regarding cardiovascular disease specifically, Black patients have had higher rates of hospitalization from myocardial infarction and greater event rates of sudden cardiac death during the COVID-19 pandemic when compared to prior studies [17,18]. Interestingly though, our study showed a decrease in admissions for non-Hispanic Black patients and no difference in rates of hospitalization for ADHF for other racial and ethnic minority groups. Non-Hispanic Black and Hispanic patients made up the majority of those admitted from neighborhoods below the average median income. The peak pandemic cohort had more patients admitted from income quartile 3, those whose median household income was average to above average when compared to the rest of the cohort. Patients from income quartile 3 tended to be White, male, and on average 2 years younger than the rest of the patients admitted in 2020. Overall, there were no major differences in comorbidities or baseline characteristics for income quartile 3 when compared to the other quartiles. It is unclear what social factors, if any, were at play that led to a higher admission rate for this income quartile.

Previous reports have differed on whether there have been higher rates of ICU admission or inpatient mortality during the COVID-19 pandemic for ADHF [3,6,19,20]. In Germany, in the largest sample, to date, of ADHF hospitalizations during the COVID-19 pandemic (N=1972), there was an increase in ICU admission rates as well as inpatient mortality [20]. In our cohort, there was also an increase in ICU admission rates for all patients admitted for ADHF. Patients from all racial and ethnic minorities as well as all neighborhood income levels saw an increase in ICU admission rates, though statistical significance of the increase in these rates was seen only in patients from income quartile 4. This income quartile made up almost 60% of the entire cohort. It is likely that our study was underpowered to find significant increases in ICU admissions among patients from lower income neighborhoods as well as those from different racial and ethnic minorities. Overall, both the prepandemic and COVID-19 pandemic cohorts had lower inpatient mortality rates than the reported national rate of 5.8% [21]. Though not reaching significance, a trend was seen for patients having higher inpatient mortality during the COVID-19 pandemic. Again, our sample size may have been too small to reach statistical significance.

Lack of follow-up after hospitalization for ADHF is associated with an increased risk of rehospitalization for patients of racial and ethnic minorities, and those from lower socioeconomic backgrounds [7,22-24]. In a face-to-face survey of patients who were recently hospitalized for ADHF, over half had a major barrier to follow-up such as no form of personal transportation [25]. Additionally, prior meta-analysis of 41 randomized controlled trials has shown that a combination of home visits, phone calls, and clinic visits were the most effective way to reduce rehospitalizations for ADHF [26]. Telemedicine may be able to circumvent some of the larger barriers to care while also supplying more effective tools for preventing rehospitalization. In our study, 7-day follow-up improved most for non-Hispanic Black patients during the pandemic. These follow-up visits were 81.6% by telemedicine. A similar trend was seen across income quartiles, with all median household income quartiles seeing improvement in 7-day follow-up. Importantly, inequities in 7-day follow-up rate in patients from non-Hispanic Black racial backgrounds as compared to those from non-Hispanic White backgrounds *decreased* during the pandemic period. Whereas in 2019, the gap was 36.6% of non-Hispanic White patients getting 7-day follow-up visits compared to 17.0% of non-Hispanic Black patients, in 2020, this gap *narrowed* to 43.6% of non-Hispanic White patients, as compared to 33% of non-Hispanic Black patients getting follow-up care. This disparity has been well reported, with Black race being associated with lower odds of early physician follow-up for not only HF but other chronic conditions as well [27-29]. It is expected that telemedicine may not only improve early physician follow-up for all patients but help reduce disparities that have long been present.

We found a striking reduction in readmission rates for patients with telemedicine follow-up compared to no follow-up. The reason for this association is not clear. It is possible that this association is confounded by an omitted variable, and that patients who can conduct a telemedicine appointment have other factors that also mitigate risk of readmission. It is also possible that during a period with limited in-person appointments, patients selected for in-person follow-up were sicker and were more likely to need readmission. However, this finding is promising, and further prospective studies should be pursued to assess if telemedicine may be a feasible option in reducing 30-day HF readmissions.

### Limitations

Our study has important limitations. While median neighborhood household income can help estimate a patient’s financial and social situation, it does not fully capture each patient’s individual socioeconomic status. Additionally, our cohort consisted of patients admitted with a primary diagnosis of ADHF, but we cannot rule out that some patients may have contracted the SARS-CoV-2 virus as an additional contributor to the higher morbidity and mortality seen during the pandemic. We did not exclude patients with SARS-CoV-2 infection because this would inaccurately reflect the true burden of HF admissions during the pandemic. Additionally, the inclusion of these patients allowed greater accuracy in assessing 7-day follow-up and 30-day readmission rates. Finally, while our hospital system has locations throughout the Chicago metropolitan area, we were unable to capture 30-day readmissions for patients who were admitted to medical centers outside of the NMHC system.

### Conclusions

Patients admitted for ADHF during the COVID-19 pandemic had higher rates of ICU admissions and a trend toward higher inpatient mortality; however, there were no major differences seen in rates between different racial, ethnic, and socioeconomic groups. Additionally, as the use of telemedicine became more ubiquitous, 7-day follow-up after hospital discharge increased for all patients and decreased disparities in follow-up. Patients who had 7-day follow-up, including telemedicine follow-up, were also less likely to be readmitted in 30 days. These findings suggest that telemedicine acts as a digital bridge rather than a digital divide in improving early follow-up, decreasing disparities in follow-up, and reducing 30-day readmissions. Future prospective randomized trials are needed to assess whether telemedicine may be a feasible tool in reducing HF readmission rates and improving access to follow-up, especially for those from marginalized communities. Further work is needed to assess whether telemedicine should remain as a viable option for the delivery of care to patients with HF during and beyond the pandemic.

## Appendix

Multimedia Appendix 1 Racial and socioeconomic differences in heart failure hospitalizations and telemedicine follow-up data set.

## Abbreviations

ADHF: acute decompensated heart failure
GWTG-HF: Get With the Guidelines—Heart Failure
HF: heart failure
ICD: International Classification of Diseases
ICU: intensive care unit
NMHC: Northwestern Memorial Healthcare
OPTIMIZE-HF: Organized Program to Initiate Lifesaving Treatment in Hospitalized Patients with Heart Failure

## Data Availability

All data generated or analyzed during this study are included in this published article or in Multimedia Appendix 1.
